# Healing of Bilateral Nipple Areolar Complex Necrosis by Secondary Intention

**DOI:** 10.7759/cureus.9025

**Published:** 2020-07-06

**Authors:** Oscar A Vazquez, Hilton Becker

**Affiliations:** 1 Surgery, Charles E. Schmidt College of Medicine, Florida Atlantic University, Boca Raton, USA

**Keywords:** plastic and reconstructive surgery, surgery, wound care, plastic surgery

## Abstract

Complete necrosis of the nipple, areola, or both is a uncommon complication of reduction mammaplasty, especially if it happens bilaterally. This case involving a young, black female illustrates that, in a large open wound of the breast, it would be ideal to leave it to heal by secondary intention as adding a skin graft would only speed up the healing process and not improve the result with the additional risk of keloid at the donor site. This open wound of the breast was treated with progressive surgical debridement while assessing the affected area in order to preserve as much tissue as possible due to the sensitive nature of the wound along with wet to dry dressing changes and antibiotic solution treatment. Our report suggests an additional approach to the standard of care involving a free flap to improve aesthetic outcomes.

## Introduction

Necrosis of the nipple areolar complex (NAC) is a rare complication of reduction mammaplasty or mastopexy. The manifestations of nipple areolar ischemia vary from spontaneous, completely reversible nipple congestion to total loss of the nipple with extensive necrosis of the adjacent breast tissue [1]. Circulatory compromise of the NAC may be due to arterial insufficiency but is more commonly caused by venous congestion [2]. The blood supply to the NAC is an important consideration intraoperatively as limitations to the NAC increase the risk of necrosis as seen in the patient presented. Once necrosis has occurred, treatment usually involves grafts. However, excessive scarring can develop as a result of aberrations of physiologic wound healing, which may be due to genetics, and can develop following any insult to the deep dermis, including burn injury, lacerations, abrasions, surgery, piercings, and vaccinations [3]. In a patient with darker skin, the risk of keloid formation from a donor-site graft, especially a patient with hypertrophic scarring on both breasts, is significant and worth considering before proceeding with further surgical intervention.

## Case presentation

**Figure 1:**
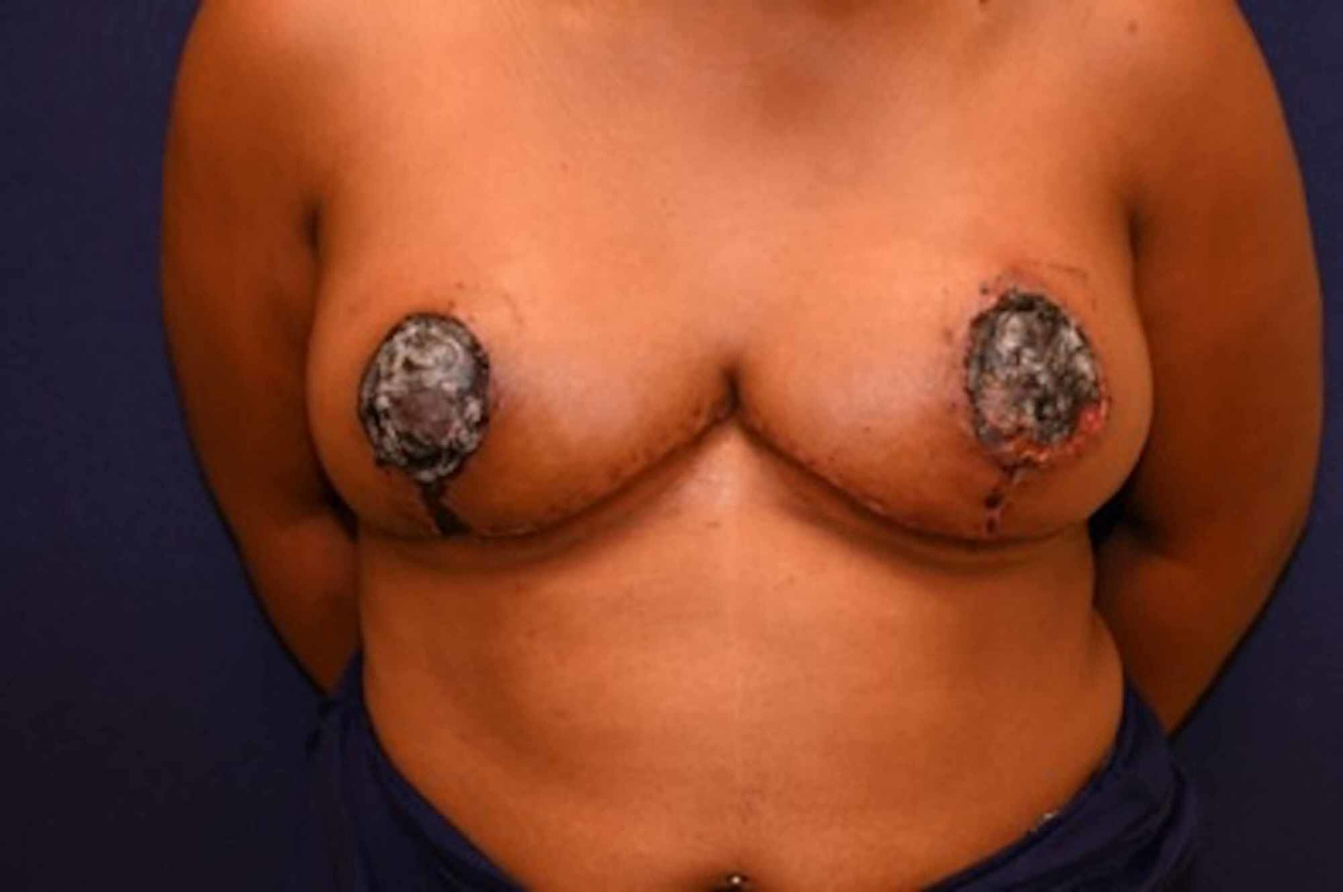
The patient presented with bilateral nipple areolar complex necrosis.

**Figure 2:**
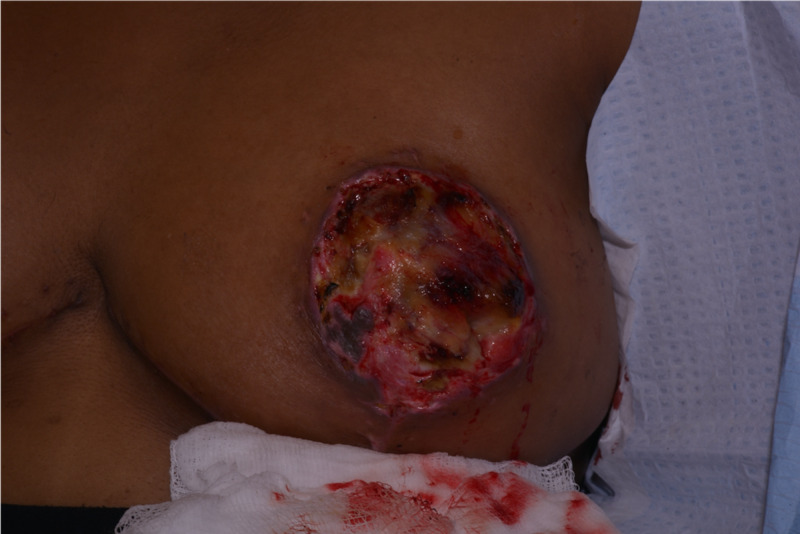
Eschar overlying areola.

A 25-year-old black female who underwent a bilateral breast reduction at another provider four weeks prior to arriving at the clinic presented with a chief concern of a foul odor coming from the areolas (Figure 1). The patient had a body mass index (BMI) of 29.5, was originally an E cup, and had a reduction of over 1 kg per side per the previous provider. She had no other past medical history, no other surgeries, reported no allergies, no current medications, and no other substances such as ethanol, smoking, or recreational drugs. Physical exam was significant only for eschar completely overlying both areolas (Figure 2). A diagnosis of bilateral NAC necrosis was made, and the subsequent treatment plan followed.

**Figure 3:**
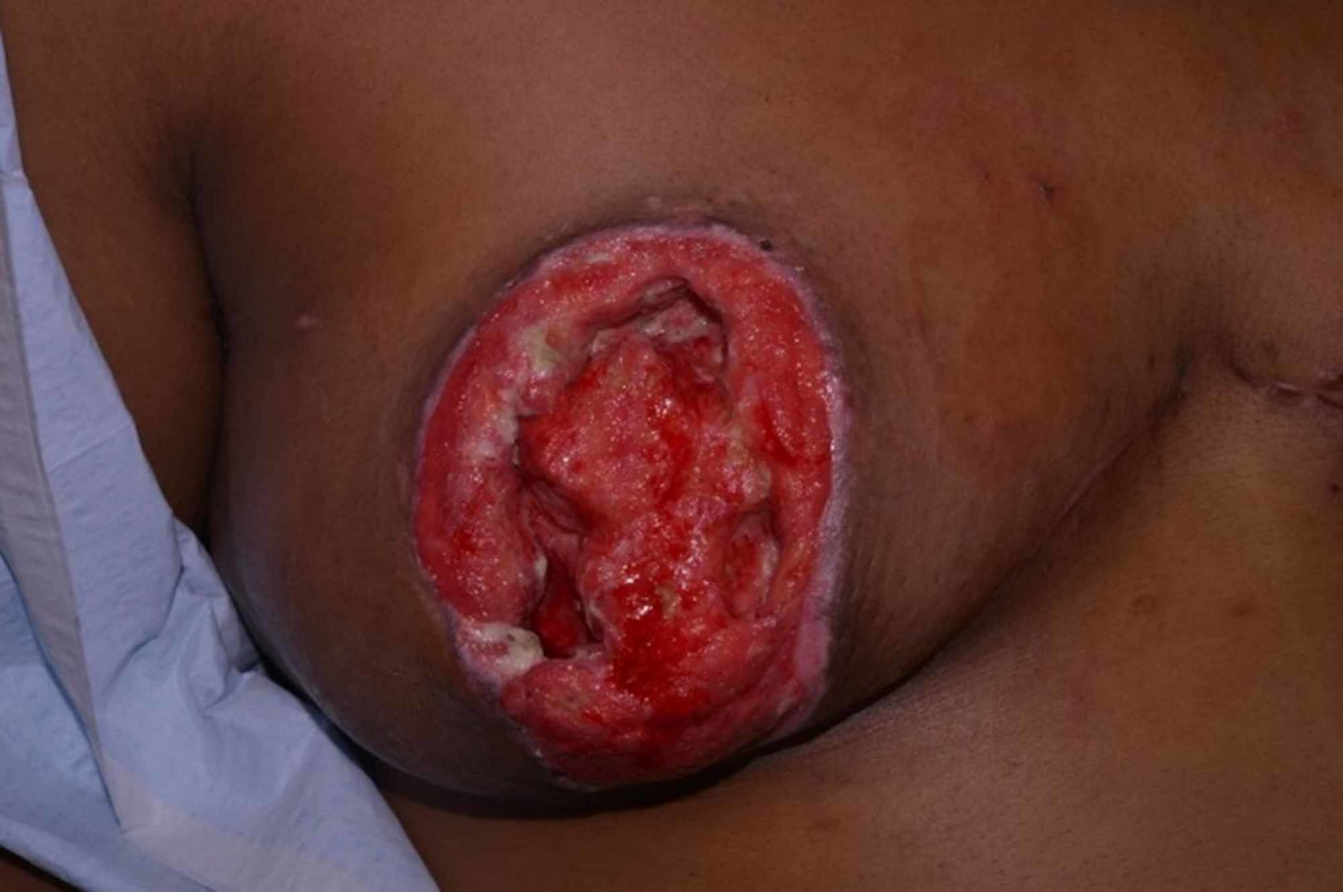
Healthy granulation tissue of the right nipple after surgical debridement.

**Figure 4:**
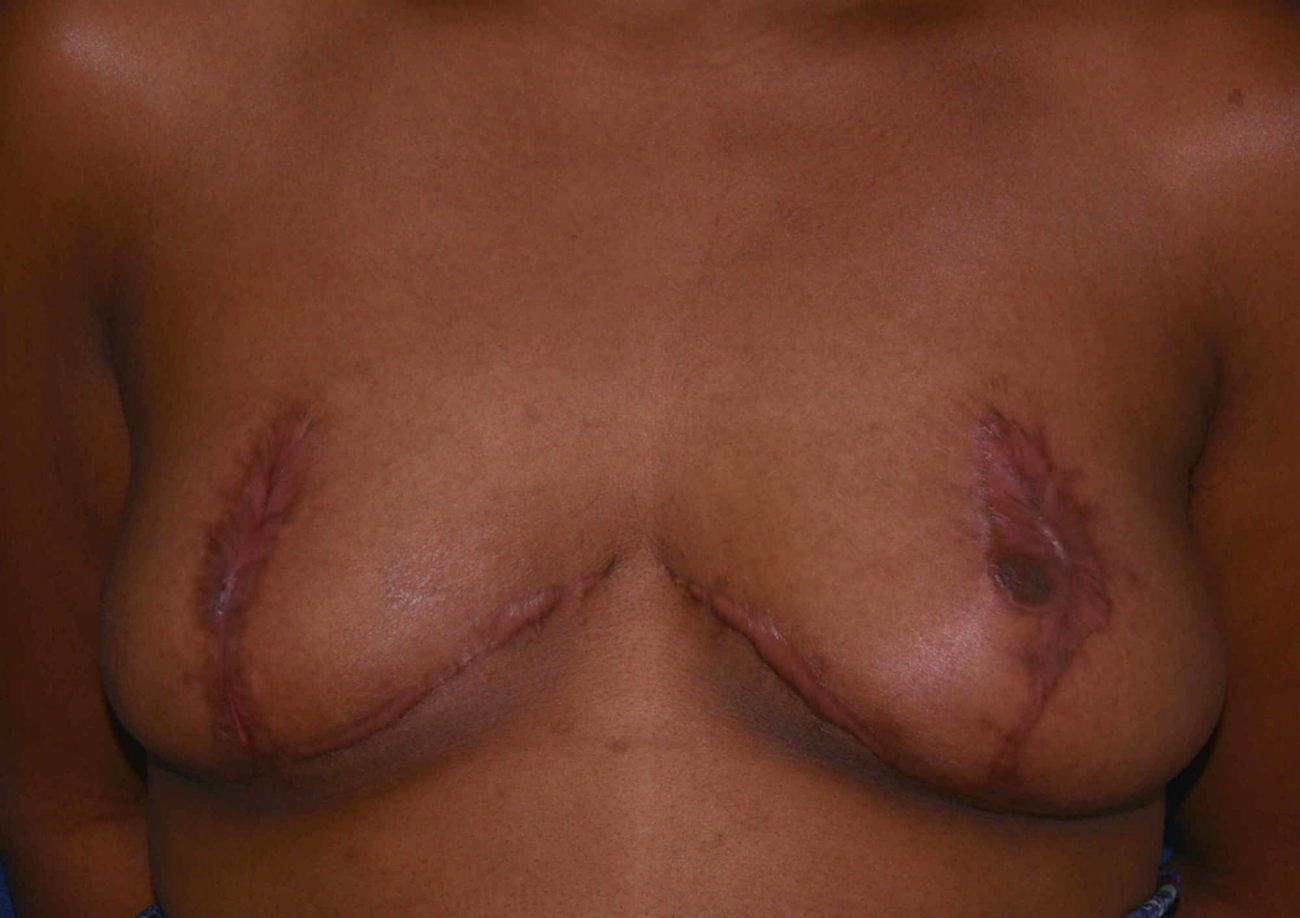
The patient 157 days after breast reduction surgery with hypertrophic mastopexy scars and healed areolar wounds.

Treatment began over the course of three months in the outpatient with partial debridement of eschar every two to three days. Further debridement was performed, excising fat necrosis extending to the muscle. This took place over the course of three weeks with patient education on wet to dry dressing application and change. At 21 days from the first encounter, debridement was combined with the breasts being packed with hypochlorous acid (HOCl) (PhaseOne, Integrated Healing Technologies, Franklin, TN) and wrapped. Wet to dry dressings were changed at follow-up appointments every two to three days by the staff and at home daily by home health care. During these appointments, healthy granulation tissue was noted (Figure 3). The patient was instructed to shower daily with antibacterial soap and cover wounds with dry gauze. Pictures were taken at 157 days post-procedure showing healed areolas (Figure 4).

## Discussion

The complication of nipple necrosis in breast reduction and mastopexy procedures has been reported in the literature to range up to 2.1% for complete NAC necrosis and 7.3% for partial NAC necrosis with a superior dermal/vertical scar [4]. Another study of 260 patients who underwent vertical reduction mammaplasty resulted in three patients with partial nipple loss due to significant excision [5]. Risk factors for these complications include obesity, diabetes, past history of poor wound healing, heavy smoking, simultaneous augmentation and mastopexy, previous radiation/chemotherapy, steroid use, previous scars around the NAC, post-bariatric surgery malnutrition, genetic predisposition to thrombosis, malignancy, and/or having large breasts as these may strain the blood supply further, which can ultimately lead to a higher rate of nipple necrosis postoperatively [6,7]. It has been shown that the blood supply to NAC is not constant, with the perforators of the internal thoracic artery (medial mammary arteries) being the most reliable source of blood supply. The NAC is also supplied by the lateral mammary branches of the lateral thoracic artery and the inferior mammary branches of the anterior intercostal arteries [2]. One study concluded that a posteroinferomedial pedicle with retained medial vertical ligament, which includes the perforating arteries of the internal thoracic artery in the pedicle, is a safer approach for breast reduction and mastopexy procedures as it ensures adequate blood supply to the nipple [8]. Recommendations for preventing NAC necrosis include maintaining a pedicle of adequate thickness, being cognizant of the length to width ratio of the pedicle, preventing kinking the blood supply when insetting the flap, and avoiding excessively tight skin closure [1].

For total necrosis of the NAC as was seen in our patient, typical treatment approaches for the nipple are composite grafts from the pulp of the toe or the earlobe, but these grafts do not match the texture or pigmentation of the nipple [9]. Composite grafts from the contralateral nipple can be the most aesthetically pleasing if there is an adequate nipple on the intact side; however, that was not an option for our patient as she had bilateral necrosis. Other approaches for reconstruction of the nipple include the star flap, the double opposing tab flap, and the double opposing periareola flap [10-13]. For reconstruction of the areola, there was also a limitation in our patient as the most natural appearing areola is usually done by using a full-thickness skin graft from the contralateral breast. Full-thickness grafts from the labia minora and the upper thigh could have been used, but they lose their pigmentation after two to three years [1].

Keloid formation is seen in individuals of all races, except albinos, but dark-skinned individuals have been found to be more susceptible to keloid formation, with an incidence of 6% to 16% in African populations [14,15]. More than 50% of all keloid patients had a positive family history of keloid scarring, and family history was strongly associated with the formation of keloid scars in multiple sites as opposed to a single anatomical site [16]. Due to the higher risk of keloid in black patients and based on the hypertrophic mastopexy scars seen in our patient, it was decided to forego harvesting a skin graft and proceeded to let these wounds heal by secondary intention. Typical treatment during the first 30 days of an open wound entails close personal contact with the patient, silver iron cream dressings with antibiotics, surgical wound debridement of nonviable surrounding tissues, and autolytic agents (Iruxol N or collagenase creams). After 30 days, weekly outpatient follow-up with photo documentation in chart is recommended along with protease-modulating agents (hydrogel and alginate creams) which provide a moist environment to speed healing [17]. Our approach combined progressive surgical debridement with consistent dressing changes along with the later addition of PhaseOne 0.025% HOCl due to its antibiotic properties [18]. Tattooing is a suitable future treatment for this patient as it can be used with or without skin grafting. Although the tattoo may fade over time, it is not difficult to restore the desired pigmentation [19].

## Conclusions

In our case, bilateral NAC necrosis was treated conservatively with delayed healing. We suggest these possible interventions as adding a skin graft would not improve the clinical outcomes, but speed up healing. Furthermore, in patients with darker skin, harvesting a skin graft may lead to inadvertent keloid scarring. Further treatment interventions for our patient include possible nipple reconstruction with fat grafting to reduce hypertrophic scarring.
